# Genistein inhibits cell invasion and motility by inducing cell differentiation in murine osteosarcoma cell line LM8

**DOI:** 10.1186/1471-2121-13-24

**Published:** 2012-09-26

**Authors:** Atsushi Nakamura, Junichi Aizawa, Kenshi Sakayama, Teruki Kidani, Tomoyo Takata, Yoshiaki Norimatsu, Hiromasa Miura, Hiroshi Masuno

**Affiliations:** 1 Department of Bone and Joint Surgery, Ehime University Graduate School of Medicine, Toon, Ehime, 791-0295, Japan; 2 Department of Medical Technology, Faculty of Health Sciences, Ehime Prefectural University of Health Sciences, Takooda, Tobe-cho, Iyo-gun, Ehime, 791-2101, Japan; 3 Musculoskeletal Tumor Surgery Center, Minami Matsuyama Hospital, Matsuyama, Ehime, 790-8534, Japan

**Keywords:** Genistein, LM8, Cell invasion, Matrix metalloproteinase-2, β-catenin, Cell differentiation

## Abstract

**Background:**

One of the problems associated with osteosarcoma is the frequent formation of micrometastases in the lung prior to diagnosis because the development of metastatic lesions often causes a fatal outcome. Therefore, the prevention of pulmonary metastases during the early stage of tumor development is critical for the improvement of the prognosis of osteosarcoma patients. In Japan, soy is consumed in a wide variety of forms, such as miso soup and soy sauce. The purpose of this study is to investigate the effect of genistein, an isoflavone found in soy, on the invasive and motile potential of osteosarcoma cells.

**Methods:**

LM8 cells were treated for 3 days with various concentrations of genistein. The effect of genistein on cell proliferation was determined by DNA measurement in the cultures and 5-bromo-2’-deoxyuridine (BrdU) incorporation study. The assays of cell invasion and motility were performed using the cell culture inserts with either matrigel-coated membranes or uncoated membranes in the invasion chambers. The expression and secretion of MMP-2 were determined by immunohistochemistry and gelatin zymography. The subcellular localization and cellular level of β-catenin were determined by immunofluorescence and Western blot. For examining cell morphology, the ethanol-fixed cells were stained with hematoxylin-eosin (H&E). The expression of osteocalcin mRNA was determined by reverse transcription-polymerase chain reaction (RT-PCR).

**Results:**

Genistein dose-dependently inhibits cell proliferation. Genistein-treated cells were less invasive and less motile than untreated cells. The expression and secretion of MMP-2 were lower in the genistein-treated cultures than in the untreated cultures. β-Catenin in untreated cells was located in the cytoplasm and/or nucleus, while in genistein-treated cells it was translocated near to the plasma membrane. The level of β-catenin was higher in genistein-treated cells than in untreated cells. Treatment of LM8 cells with genistein induced morphological changes, markedly decreased the formation of multilayer masses of cells, and markedly increased the expression of osteocalcin mRNA.

**Conclusions:**

Genistein decreased invasive and motile potential by inducing cell differentiation in LM8 cells. Genistein may be useful as an anti-metastatic drug for osteosarcoma through its differentiation-inducing effects.

## Background

Osteosarcoma occurs mainly in the metaphyseal region of the long bones of young people. The most common sites affected are the distal femur, the proximal tibia and the proximal humerus [1,2]. Osteosarcoma grows aggressively at the primary site and often develops micrometastases in the lung prior to diagnosis. The primary treatment of osteosarcoma is the complete removal of tumor by wide excision and aggressive adjuvant chemotherapy [3]. Despite progress in chemotherapy, the development of metastatic tumors in the lung often has a fatal outcome [2,4,5]. Therefore, the prevention of pulmonary metastases during the early stage of tumor development is critical for the improvement of the prognosis of osteosarcoma patients.

Metastasis is composed of multiple sequential steps, including cell detachment from the primary tumor, cell motility and invasion, angiogenesis, intravasation into blood, and extravasation into distant organs. A number of factors, such as MMP-2 and vascular endothelial growth factor (VEGF) [6-10], are involved in tumor growth, invasion, and metastasis. An LM8 osteosarcoma cell line with high metastatic potential to the lung was established from murine Dunn osteosarcoma cells, which did not develop pulmonary metastasis when implanted subcutaneously (s.c.) into the backs of C3H mice [6]. LM8 cells have been used as an excellent tool for studying inhibitory agents against pulmonary metastasis [7,8]. Previously, we reported that treatment of nude mice implanted s.c. with LM8 cells into the backs with troglitazone, a peroxisome proliferator-activated receptor-γ (PPARγ) ligand, decreased the expression of MMP-2 and VEGF within the primary tumor and inhibited the development of pulmonary metastasis [8].

Soy contains phytoestrogen isoflavones (e.g. genistein, daidzein). In Japan, soy is widely consumed in a wide variety of forms, such as non-fermented foods (dried and green soybeans, tofu, soy milk) and fermented foods (miso, soy sauce). An epidemiological study has shown that consumption of miso soup is inversely associated with the risk of breast cancer [11]. Several experimental studies have shown that genistein inhibits the growth of tumors [12-14]. In the present study, we performed *in vitro* experiments to analyze the effect of genistein on the growth, invasion, and motility of LM8 cells. Since β-catenin is associated with tumor cell growth, invasion, motility, and metastasis [15-17], the effect of genistein on the cellular level and subcellular localization of β-catenin was also examined.

## Results

### Effect of genistein on cell proliferation and DNA replication

**Figure 1:**
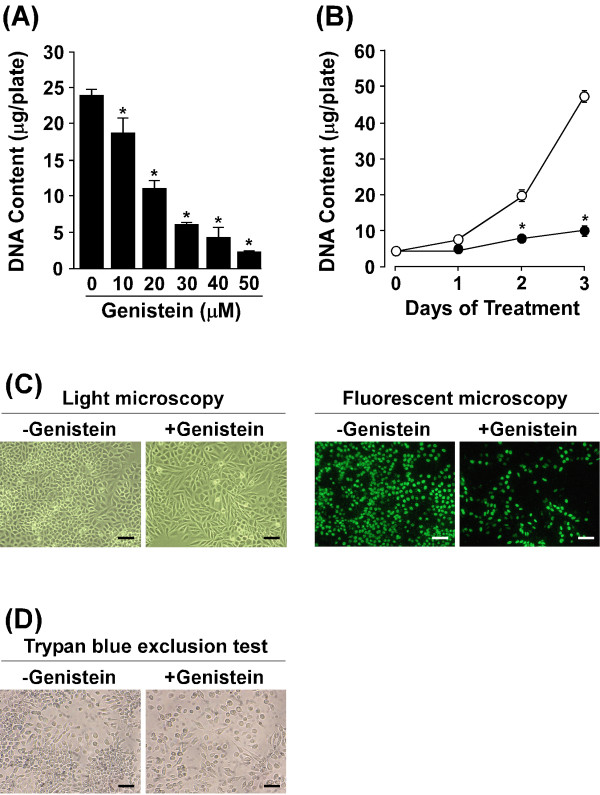
**Effect of genistein on cell proliferation and DNAreplication.** (**A**) LM8 cells were treated for 3 days with 0–50 μM genistein, and the DNA content of the cultures was measured. *p < 0.01 (compared with the values for the untreated cultures). (**B**) LM8 cells were treated without (open circle) or with (filled circle) 50 μM genistein, the DNA content of the cultures was measured at the indicated intervals. *p < 0.01 (compared with the values for the untreated cultures). (**C**) LM8 cells were treated for 3 days without (left panel) or with (right panel) 50 μM genistein, and immunofluorescence staining of BrdU incorporated into DNA was performed. Both set of images are of the same field of view. Scale bar: 50 μm. (**D**) LM8 cells were treated for 3 days without (left panel) or with (right panel) 50 μM genistein and stained with trypan blue. Scale bar: 50 μm.

We first evaluated the anti-proliferative effect of genistein against LM8 cells. For this, LM8 cells were treated for 3 days with genistein at the indicated concentrations and the DNA content of the cultures was measured. The untreated cultures (i.e., genistein was absent during the 3-day treatment period) contained 23.9 μg/35-mm plate of DNA. Genistein dose-dependently decreased the DNA content of the cultures (Figure 1A). Genistein at 50 μM decreased the DNA content by 91%. Figure 1B shows the time course of the genistein-induced changes in the DNA content. In the untreated and genistein-treated cultures, the DNA content increased during the 3-day treatment period. On day 1, there was no difference in the DNA content between the two cultures. On days 2 and 3, the DNA content of the genistein-treated cultures was significantly lower than that of the untreated cultures.

LM8 cells were incubated with 5-bromo-2’-deoxyuridine (BrdU) during the last 90 min of the 3-day treatment period to label DNA synthesis (Figure 1C). In the untreated and genistein-treated cultures, positive BrdU immunofluorescence staining was observed in the nucleus. The BrdU-labeling index of the genistein-treated cultures (32.4 ± 3.8%) was significantly (p < 0.01) lower than that of the untreated cultures (56.4 ± 3.0%).

To examine the effect of genistein on cell viability, the trypan blue exclusion test was performed (Figure 1D). In the untreated and genistein-treated cultures, cells that attached to the bottom of the plates excluded trypan blue.

### Effect of genistein on subcellular localization and cellular level of β-catenin

**Figure 2:**
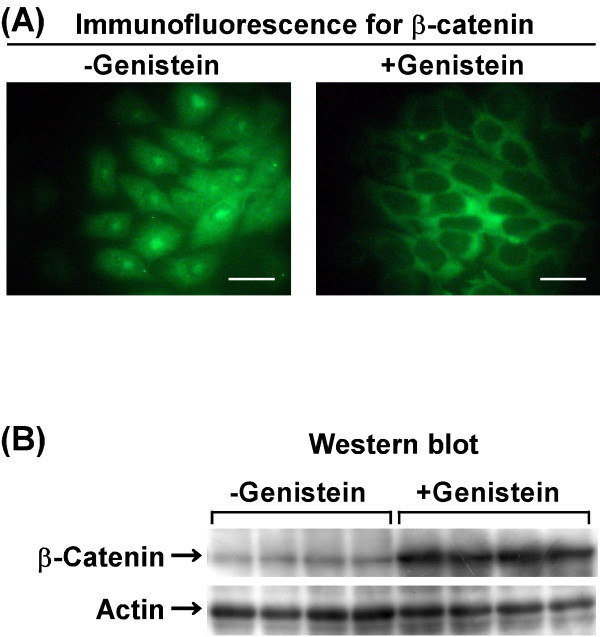
**Effect of genistein on subcellular localization and cellularlevel of β-catenin.** (**A**) LM8 cells were treated for 3 days without (left panel) or with (right panel) 50 μM genistein, and immunofluorescence staining of β-catenin was performed. Scale bar: 50 μm. (**B**) LM8 cells were treated for 3 days without or with 50 μM genistein, and the expression of β-catenin and actin was analyzed by Western blot.

The subcellular localization of β-catenin was examined by immunofluorescence. In the untreated cultures, positive β-catenin immunofluorescence staining was observed in the cytoplasm and/or nucleus and was not observed at the plasma membrane (Figure 2A, left panel). In the genistein-treated cultures, positive β-catenin immunofluorescence staining shifted in localization near to the plasma membrane and was not observed in the nucleus (Figure 2A, right panel).

The cellular level of β-catenin was examined by Western blot. The intensity of the band corresponding to β-catenin was stronger in the genistein-treated cultures than in the untreated cultures (Figure 2B), indicating that genistein increased the cellular level of β-catenin.

### Effect of genistein on invasion, motility, MMP-2, and alkaline phosphatase (ALP)

**Figure 3:**
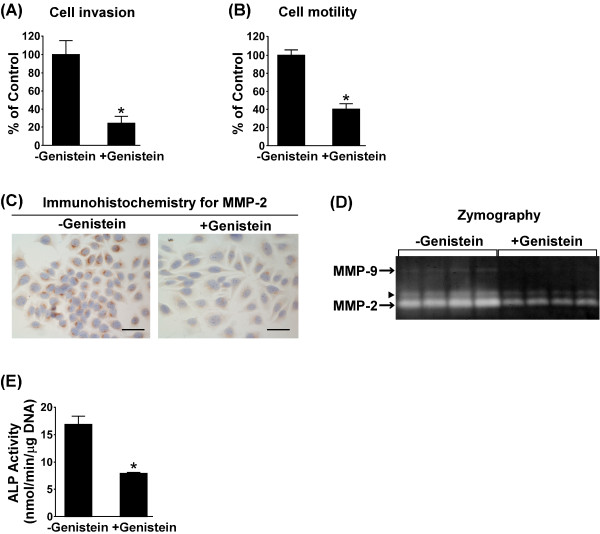
**Effect of genistein on invasion, motility, MMP-2, andALP.** (**A** and **B**) LM8 cells were treated for 3 days without or with 50 μM genistein and harvested by trypsinization. Cell invasion and motility assays were performed using either matrigel-coated membranes (**A**) or uncoated membranes (**B**). *p < 0.01 (compared with the values for untreated cells). (**C**) LM8 cells were treated for 3 days without (left panel) or with (right panel) 50 μM genistein, and immunohistochemical staining of MMP-2 was performed. Scale bar: 50 μm. (**D**) LM8 cells were treated for 3 days without or with 50 μM genistein. The activity of MMP-2 secreted into conditioned media during the last 24 h of the 3-day treatment was assayed by gelatin zymography. Arrowhead shows the preform of MMP-2. (**E**) LM8 cells were treated for 2 days without or with 50 μM genistein, and cellular ALP activity was measured. *p < 0.01 (compared with the values for untreated cells).

LM8 cells, which had been treated for 3 days without or with 50 μM genistein, were harvested by trypsinization, and cell invasion and cell motility assays were performed. When experiments were performed using matrigel-coated membranes, the absorbance of the dye extracted from genistein-treated cells was 24% of that extracted from untreated cells (Figure 3A). This indicates that genistein-treated cells were less invasive than untreated cells. When experiments were performed using uncoated membranes, the absorbance of the dye extracted from genistein-treated cells was 40% of that extracted from untreated cells (Figure 3B). This indicates that genistein-treated cells were less motile than untreated cells.

Since MMP-2 degrades type IV collagen, which is present in vascular and subepithelial basement membranes, and plays a pivotal role in cell invasion [9], the expression of MMP-2 was examined by immunohistochemical staining (Figure 3C). In both untreated and genistein-treated cells, positive MMP-2 immunostaining was observed in the cytoplasm. The intensity of immunostaining was weaker in genistein-treated cells than in untreated cells, thus indicating that genistein-treated cells expressed less MMP-2 than untreated cells.

The activity of MMP-2 secreted into conditioned media during the last 24 h of the 3-day treatment period was assayed by gelatin zymography (Figure 3D). MMP-2 was secreted from both untreated and genistein-treated cells; however, the activity of MMP-2 secreted from genistein-treated cells was lower than that secreted from untreated cells. Low MMP-9 activity was detected in conditioned media from untreated cells, but not genistein-treated cells.

The ALP activity in LM8 cells is higher than that in Dunn cells [6], suggesting that the ALP activity in osteosarcoma cells may be associated with metastatic potential. We treated LM8 cells without or with 50 μM genistein for 2 days and measured cellular ALP activity (Figure 3E). The ALP activity in genistein-treated cells (7.9 ± 0.2 nmol/min/μg DNA) was significantly lower than that in untreated cells (16.9 ± 1.5 nmol/min/μg DNA).

### Does genistein induce the differentiation of LM8 cells?

**Figure 4:**
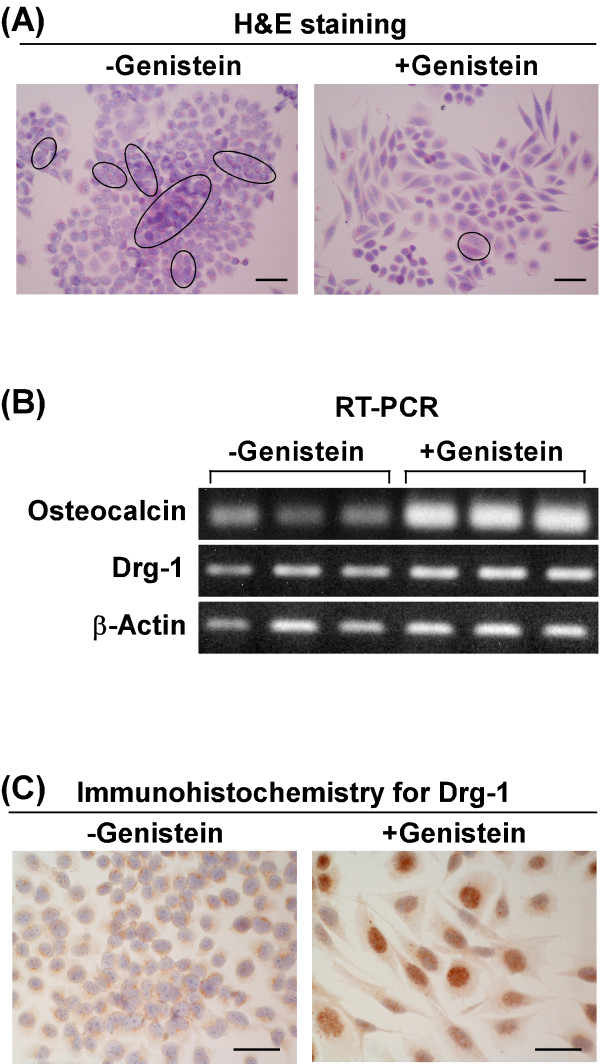
**Effect of genistein on differentiation of LM8 cells.** (**A**) LM8 cells were treated for 3 days without (left panel) or with (right panel) 50 μM genistein, fixed with ethanol, and stained with H&E. Scale bar: 50 μm. (**B**) LM8 cells were treated for 3 days without or with 50 μM genistein, and the expression of mRNAs of osteocalcin, Drg-1, and β-actin was examined by RT-PCR. (**C**) LM8 cells were treated for 3 days without (left panel) or with (right panel) 50 μM genistein, and immunohistochemical staining of Drg-1 was performed. Scale bar: 50 μm.

We stained ethanol-fixed LM8 cells with hematoxylin-eosin (H&E) and observed cell morphology. In the untreated cultures, the majority of cells (92.0 ± 2.0%) were cuboidal in shape with filopodial and lamellipodial structures surrounding the cell surface (Figure 4A, left panel). These cells grew to form numerous multilayer masses even at low cell density (in ellipse in Figure 4A). The genistein-treated cultures contained two morphologically different cell types; one was cuboidal in shape and the other was spindle-shaped, which became more flattened with clear cell-cell margins (Figure 4A, right panel). The ratio of the number of spindle-shaped cells to the total number of cells was 46.4 ± 2.9%. Multilayered cells were markedly decreased in the genistein-treated cultures. These findings suggest that genistein may induce the differentiation of LM8 cells.

To confirm this, we examined the expression of mRNAs of osteocalcin, which is a marker of mature osteoblasts [18], and differentiation-related gene-1 (Drg-1), which is associated with the induction of differentiation in tumors [19], by reverse transcription-polymerase chain reaction (RT-PCR) (Figure 4B). Untreated cells expressed a low level of osteocalcin mRNA, while genistein-treated cells expressed a markedly high level of osteocalcin mRNA. Untreated cells expressed a significant level of Drg-1 mRNA. The level of Drg-1 mRNA in genistein-treated cells was similar to that in untreated cells.

The subcellular localization of Drg-1 was examined by immunohistochemical staining. In untreated cells, positive Drg-1 immunostaining was observed in the cytoplasm (Figure 4C, left panel), while in genistein-treated cells, it was mostly observed in the nucleus (Figure 4C, right panel).

## Discussion

Since genistein has been reported to inhibit tumor growth [12-14], we first examined the effect of genistein on the proliferation of LM8 cells. The results of BrdU incorporation into DNA and DNA measurement revealed that genistein inhibited DNA replication and cell proliferation in cultures of LM8 cells (Figure 1A and 1C). Of course, there is concern that this inhibition could be due to a cytotoxic effect of genistein on LM8 cells. However, this does not appear to be the case because cells did not detach from the bottom of the plates during the 3-day treatment period. Moreover, the results of the trypan blue exclusion test showed that genistein-treated cells that attached to the bottom of the plates were viable cells (Figure 1D). Genistein at a higher concentration (100 μM) also inhibited the proliferation of LM8 cells without affecting cell viability (data not shown). These findings indicate that genistein-induced inhibition of cell proliferation was not due to a cytotoxic effect. There are reports on the effect of genistein on the proliferation of normal cells. In cultures of 3T3-L1 fibroblasts, genistein at 50 and 100 μM inhibits cell proliferation [20]. In cultures of endothelial cells, genistein at lower concentrations (0.001-10 nM) stimulates cell proliferation, while at a higher concentration (1 μM) this chemical inhibits it [21].

When LM8 cells are implanted s.c. into the backs of mice, a 100% pulmonary metastatic rate is observed within 4 weeks [6-8]. The initial step of metastasis is cell detachment from the primary tumor. Since β-catenin is essential for cadherin-mediated cell-cell adhesion, reduced expression of β-catenin and cadherins at the cell surface is associated with tumor metastasis [22,23]. Loss of membranous β-catenin occurs commonly in primary colorectal cancers with metastatic potential and in the corresponding colorectal liver metastases [23]. Our results of immunofluorescence showed loss of membranous β-catenin in LM8 cells (Figure 2A, left panel), indicating that cell-cell adhesiveness may be reduced in LM8 cells.

Genistein affects the subcellular localization and expression level of β-catenin in normal cells. For example, treatment of mouse HC11 mammary epithelial cells with genistein increases membranous β-catenin [24]. In mammary glands of young female rats fed a genistein-containing diet, β-catenin is predominantly localized to ductal epithelial cell membranes [25] and the level of this protein is higher compared with those fed a control diet [24]. Therefore, we examined whether genistein affects the subcellular localization and cellular level of β-catenin in LM8 cells. In genistein-treated cells, immunofluorescence staining of β-catenin was observed near the plasma membrane (Figure 2A, right panel). The results of Western blot showed that genistein-treated cells contained higher levels of β-catenin than untreated cells (Figure 2B). These findings suggest that genistein may promote intracellular adhesion of LM8 cells. This is the first report to focus on the subcellular localization and cellular level of β-catenin following genistein treatment in osteosarcoma cells.

The next step of metastasis is invasion of tumor cells into basement membranes, which involves distinct events, such as cell motility and MMP expression. Invasive tumors exhibit active migration and high levels of MMPs [9]. LM8 cells have been reported to exhibit higher invasive potential, higher MMP-2 activity, and higher ALP activity than Dunn cells with no metastatic potential [6]. In patients with osteosarcoma, high levels of ALP activity in serum are associated with a poor clinical outcome [3]. In the present study, we found that genistein-treated cells were less invasive and less motile (Figure 3A and 3B), exhibited lower expression and secretion of MMP-2 (Figure 3C and 3D), and exhibited lower ALP activity (Figure 3E) compared with untreated cells. Taken together, our findings suggest that genistein-treated cells might lose metastatic potential.

When β-catenin is translocated to the nucleus, it activates target genes, such as cyclin D1 and c-*myc*, and promotes the growth of tumor cells [15,26,27]. In intestinal-type gastric cancer, β-catenin in the nucleus not only plays a role in early tumor growth, but it also functions in initiation of invasive processes [27]. In osteosarcoma that has metastasized to the lung, β-catenin is detected in the cytoplasm and/or nucleus, whereas in primary osteosarcoma that has not metastasized for more than five years, it is not detected in the cytoplasm and/or nucleus [16], indicating that the presence of β-catenin in the cytoplasm and/or nucleus is a marker of metastatic potential of osteosarcoma to the lung. Our results of immunofluorescence showed that β-catenin was present in the cytoplasm and/or nucleus of LM8 cells (Figure 2A, left panel) and that after treatment of LM8 cells with genistein, β-catenin was translocated near to the plasma membrane and was not found in the nucleus (Figure 2A, right panel). This translocation of β-catenin may result in decreases in proliferative rate and invasive potential of genistein-treated cells. A similar translocation of β-catenin from the nucleus to the plasma membrane has been reported in human HT-29 colon cancer cells treated with thiazolidinedione, a PPARγ ligand [17].

Treatment of LM8 cells with genistein induced morphological changes (Figure 4A) and markedly increased the level of osteocalcin mRNA (Figure 4B). Moreover, this treatment markedly decreased the formation of multilayer masses (Figure 4A), suggesting that genistein-treated cells grew with contact inhibition. Contact inhibition is a characteristic of normal cells grown on plastic culture plates, which is lost in cancerous cells [28]. On the basis of these findings, we concluded that genistein induced the differentiation of LM8 cells. There are a few reports on the association of genistein with cell differentiation. Treatment of mouse B16 melanoma cells with genistein induces morphological changes characteristic of differentiation, such as enlargement of the soma and nuclei together with the formation of dendritic-like structures [12]. Treatment of human MG 63 osteosarcoma osteoblasts with genistein increases the level of collagen and stimulates the formation of calcification foci [13].

Drg-1 is expressed at lower levels in tumors than in normal tissue [19,29]. Overexpression of *Drg-1* gene in human SW620 metastatic colon cancer cells induces morphological changes that are similar to differentiation-specific changes induced by known differentiation reagents such as tributyrin and a ligand of retinoid X receptor LG268 [30], thus indicating that Drg-1 plays a role in differentiation of tumor cells. However, there is a contradictory report on the involvement of Drg-1 in cell differentiation. Human hepatocellular carcinomas express a higher level of Drg-1 than non-tumor liver or cirrhotic and benign liver lesions [31]. Moreover, hepatocellular carcinomas that are moderately and poorly differentiated express a higher level of Drg-1 than those that are well differentiated [31]. The positive or negative association of Drg-1 with cell differentiation depends on tumor types. Therefore, we examined the effect of genistein on the expression of Drg-1 mRNA by RT-PCR. LM8 cells significantly expressed Drg-1 mRNA. Genistein did not increase the expression of Drg-1 mRNA in LM8 cells (Figure 4B). Thus, the expression of Drg-1 in LM8 cells appears not to be associated with the induction of cell differentiation.

Drg-1 is located in the cytoplasm of human EJ bladder carcinoma cells [29], human hepatocellular carcinoma cells [31], and human prostate cancer cells [32]. Our results of immunohistochemical staining also reveal that Drg-1 in LM8 cells was located in the cytoplasm (Figure 4C, left panel). After treatment of LM8 cells with genistein, Drg-1 mostly shifted in localization from the cytoplasm to the nucleus (Figure 4C, right panel). A study with human U2OS osteosarcoma cells expressing Drg-1 small interfering RNA showed that Drg-1 is associated with the production of osteocalcin [33]. Taken together, our findings suggest that Drg-1 in the nucleus of genistein-treated cells may play a role in the expression of osteocalcin mRNA.

In postmenopausal Japanese women [11] and Asian-American women [34], the risk of breast cancer is inversely associated with isoflavone intake during adult life. Asian-American women who are high consumers of isoflavone (>12.68 mg/1,000 kcal) show a significantly reduced risk of breast cancer compared with those who are low consumers (≤1.79 mg/1,000 kcal) [34]. However, isoflavones at the levels commonly consumed by non-Asian US women (an average intake equivalent to less than one serving of tofu per week) have little effect on breast cancer risk [35]. In mice implanted with human 253J B-V bladder cancer cells, dietary genistin (0.14% of the diet), the natural form of genistein in soy, inhibits tumor growth at the primary site and pulmonary metastasis [14]. In mice implanted with human PC3-M prostate cancer cells, dietary genistein (100 and 250 mg/kg diet) inhibits pulmonary metastasis without affecting tumor growth at the primary site [36].

## Conclusions

Genistein induced cell differentiation and translocated β-catenin near to the plasma membrane, resulting in lowering of the proliferative rate and invasive potential of LM8 cells. Genistein may be useful as an anti-metastatic drug for osteosarcoma through its differentiation-inducing effects.

## Methods

### Cell culture

Genistein (Sigma-Aldrich, St. Louis, MO) was dissolved in dimethyl sulfoxide. LM8 cells (RBRC-RCB1450; Riken BRC Cell Bank, Ibaraki, Japan) at a concentration of 1.25 × 10^3^ cells/cm^2^ were seeded on a 35 mm plate in the culture medium, which contained 10% fetal bovine serum (FBS), 100 units/ml penicillin, and 100 μg/ml streptomycin in Dulbecco’s modified Eagle’s medium (DMEM). After 24 h of seeding, the medium was replaced with culture medium containing genistein at the indicated concentrations. Then, cells were incubated for 1–3 days.

For trypan blue exclusion test, cells were treated for 3 days without or with 50 μM genistein, washed twice with phosphate-buffered saline (PBS), stained for 1 min with 0.25% trypan blue in PBS, and washed 3 times with PBS for phase-contrast microscopy analysis (magnification: ×100).

### Measurements of DNA and ALP activity

For DNA measurement, cells were treated for 1–3 days without or with genistein (10–50 μM), harvested in 0.3 ml of solution A (10 mM Tris, 0.1% Triton X-100, pH 7.5), sonicated briefly at 0°C, and centrifuged. DNA in the supernatant was measured fluorometrically by the method of Hinegardner [37] using calf thymus DNA as a standard. For measurement of ALP activity, cells were treated for 2 days without or with 50 μM genistein. The ALP activity in the supernatant of cell homogenate was measured using a kit for ALP (Alkaline phospha B-test; Wako Pure Chemical Industries Ltd., Osaka, Japan) according to the manufacturer’s instructions. The results are expressed as the means ± SD of four plates.

### Cell invasion and motility assays

Cell invasion and motility were determined using the cell culture inserts with either matrigel-coated membranes (6 wells, 8 μm pore size; BD Biosciences, Franklin Lake, NJ) or uncoated membranes (12 wells, 8 μm pore size; BD Biosciences) in the invasion chambers as described previously [8]. Briefly, the chambers were assembled using the inserts with either matrigel-coated membranes or uncoated membranes and DMEM containing 10% FBS as a chemoattractant in the lower compartment. LM8 cells, which had been treated without or with 50 μM genistein for 3 days, were harvested by trypsinization, and suspended in DMEM containing 0.1% bovine serum albumin (BSA). The cells (5 × 10^5^ cells/2 ml for invasion assay or 2.5 × 10^5^ cells/ml for motility assay) were added to the inserts. The assembled chambers were incubated for 48 h or 24 h at 37°C. After removal of non-migrating cells on the upper surface of the membrane by wiping with a cotton swab, the cells on the bottom surface of the membrane were fixed with 100% ethanol for 30 sec and stained with toluidine blue for 10 min. The membrane was washed with ethanol and dried. The dye was dissolved with 10% acetic acid and quantitated by measuring the absorbance at 590 nm. The results are expressed as the means ± SD for six membranes.

### Immunofluorescence and immunohistochemical staining

LM8 cells (1.19 × 10^3^ cells/cm^2^) were incubated for 24 h on a 2-well chamber slide (Nalge Nunc International, Osaka, Japan). Then, cells were treated for 3 days without or with 50 μM genistein. The cells were incubated with 30 μM BrdU (Wako Pure Chemical Industries) during the last 90 min of the 3-day treatment period, fixed in 70% ethanol for 30 min, incubated in 100% ethanol for 10 min, treated with 1.5 N HCl for 30 min, and blocked with 0.5% Tween 20 for 5 min. Thereafter, cells were incubated for 1 h with a mouse monoclonal anti-BrdU antibody (1:15 dilution; Dako Japan, Inc., Tokyo, Japan) followed by 1-h incubation with a fluorescein isothiocyanate (FITC)-labeled anti-mouse IgG (1:20 dilution; Zymed Laboratories, Inc., San Francisco, CA) in the dark. Cells were then mounted in fluorescence mounting medium (Dako Japan) for fluorescent microscopy analysis (magnification: × 100). Five fields of the culture were randomly photographed. The BrdU-labeling index was calculated by dividing the number of BrdU-positive cells by the total number of cells (170–795 cells/a field). The results are expressed as the mean ± SD for five determinations. The antibodies used for immunofluorescence and immunohistochemical staining were diluted with PBS containing 1% BSA.

For immunofluorescence staining of β-catenin, cells were treated for 3 days without or with 50 μM genistein, fixed with ethanol as described above, and incubated for 1 h with a rabbit polyclonal antibody to β-catenin (1:15 dilution; Santa Cruz Biotechnology, Inc., Santa Cruz, CA) followed by 1-h incubation with an FITC-labeled anti-rabbit IgG (1:20 dilution; Santa Cruz Biotechnology) in the dark. Cells were then mounted in fluorescence mounting medium for fluorescent microscopy analysis (magnification: × 400). For immunohistochemical staining of MMP-2 and Drg-1, the ethanol-fixed cells were incubated for 1 h with rabbit polyclonal antibodies to either MMP-2 or Drg-1 (1:15 dilution; Santa Cruz Biotechnology) followed by 1-h incubation with horseradish peroxidase (HRP)-conjugated ENVISION+ (Dako Japan). The positive cells were visualized by adding diaminobenthidine (DAB; Dako Japan). The nuclei were counterstained with hematoxylin. Cells were then mounted in glycergel (Dako Japan) for light microscopy analysis (magnification: × 400).

### H&E staining

LM8 cells were treated for 3 days without or with 50 μM genistein, fixed with ethanol as described above, and stained with H&E. Cells were then mounted in glycergel for light microscopy analysis (magnification: × 200). Five fields of the culture were randomly photographed. The ratio of the number of spindle-shaped cells to the total number of cells (102–156 cells/a field) was estimated, with the exception of multilayered cells. The results are expressed as the mean ± SD for five determinations.

### Western blot analysis

LM8 cells (1.25 × 10^3^ cells/cm^2^) were incubated for 24 h on a 60 mm plate. Then, cells were treated for 3 days without or with 50 μM genistein, harvested in 0.6 ml of solution A containing a protease inhibitor cocktail (1:100 dilution; Calbiochem-Novabiochem Co., La Jalla, CA), sonicated briefly at 0°C, and centrifuged. The same amount of supernatant protein (16.5 μg/lane) was separated by sodium dodecyl sulphate-polyacrylamide gel electrophoresis (SDS-PAGE, 7.5% acrylamide gel) and transferred onto a PVDF membrane (GE Healthcare UK Ltd. Buckinghamshire HP7 9NA, England). The membrane was incubated for 1 h with rabbit polyclonal antibodies to either β-catenin or actin (1:2,000 dilution; Santa Cruz Biotechnology) followed by 1-h incubation with a HRP-conjugated anti-rabbit IgG (1:25,000 dilution; GE Healthcare). Blots were visualized using the ECL Advance Western Blotting Detection Kit (GE Healthcare) according to the manufacturer’s instructions. The experiment was performed using four independent samples for each treatment.

### Assay of MMP-2 by gelatin zymography

LM8 cells were treated for 2 days without or with 50 μM genistein, washed 3 times with PBS, and incubated with FBS-free DMEM for 1 h. The medium was then replaced with FBS-free DMEM without or with 50 μM genistein, and the cells were incubated for 24 h. The conditioned media were filtered through 0.2 μm filters. The activity of MMP-2 in conditioned media was assayed by gelatin zymography as described previously [8]. The experiment was performed using four independent samples for each treatment.

### Total RNA isolation and RT-PCR

Total RNA was isolated from a 60 mm plate using a GenElute Mammalian Total RNA Kit (Sigma-Aldrich) according to the manufacturer’s instructions. Total RNA (1 μg) was treated with DNase I (Sigma-Aldrich) and then reverse-transcribed using the Transcriptor First Strand cDNA Synthesis Kit (Roche, Indianapolis, IN) according to the manufacturer’s instructions. 1 μl of cDNA was subjected to PCR in a final volume of 25 μl containing 1 × PrimeSTAR™ reaction buffer, 0.2 mM dNTPs, 1 mM MgCl_2_, 0.4 μM of each primer, and 0.625 U of PrimeSTAR™ HS DNA Polymerase (Takara Bio Inc., Otsu, Japan). The primers for mouse osteocalcin [38], mouse Drg-1 (from NCBI/Primer-BLAST), and mouse β-actin [39] are listed in Table 1. The PCR condition was as follows: 10 sec at 98°C (denaturation), 10 sec at 56°C (annealing), and 30 sec at 72°C (extension) for osteocalcin and β-actin; 10 sec at 98°C, 10 sec at 60°C, and 30 sec at 72°C for Drg-1. The PCR products were separated on 2% agarose gels and visualized by ethidium bromide staining. The experiment was performed using three independent RNA samples for each treatment.

**Table 1:** **Primers used for RT-PCRanalysis**

	**Primers**	**Product (bp)**	**Cycles**
Osteocalcin		184	30
forward	5’-GGGCAATAAGGTAGTGAACAG-3’		
reverse	5’-GCAGCACAGGTCCTAAATAGT-3’		
Drg-1		433	31
forward	5’-CGCTGAGGTGAAGCCTCTGGTG-3’		
reverse	5’-AGGGTTGTTGAGTGCGAAGCGG-3’		
β-actin		275	22
forward	5’-CAGGAGATGGCCACTGCCGCA-3’		
reverse	5’-AGGGTTGTTGAGTGCGAAGCGG-3’		

### Statistical analyses

Significant differences among multiple independent groups were statistically evaluated using one-way ANOVA and subsequent comparisons were made with the Tukey-Kramer test. Significant differences between 2 independent groups were analyzed with Student’s *t*-test. For all statistical analyses, the criterion for significance was p < 0.05.

## Competing interests

The authors declare that they have no competing interests.

## Authors’ contributions

AN participated in the design, and performed the bulk of experiments. JA participated in the proliferation, invasion, and migration assays. KS and TK participated in the experimental design, coordination and the corrections of the manuscript. TT participated in the RT-PCR experiments. YN participated in the immunohistochemical experiments. HM (H. Miura) participated in the experimental design, coordination and the corrections of the manuscript. HM (H. Masuno) was responsible for the experimental design, statistical analysis of the data, drafting of the manuscript and conceiving this study. All authors read and approved the final manuscript.
